# Cluster of pigmented macules in a pediatric patient

**DOI:** 10.1016/j.jdcr.2022.08.055

**Published:** 2022-09-08

**Authors:** Krystina Khalil, Claudia Green, Derrek Giansiracusa, Gabriella Vasile, Eduardo Weiss

**Affiliations:** a LECOMT/Larkin Community Hospital, Palm Springs Campus, Hialeah, Florida; b Lake Erie College of Osteopathic Medicine, Bradenton, Florida; c Hollywood Dermatology and Cosmetic Surgery, Hollywood, Florida

**Keywords:** agminated lentiginosis, hyperpigmentation, lentiginous mosaicism, neurofibromatosis, nevus spilus, partial unilateral lentiginosis, pigmented lesions, NF1, neurofibromatosis type 1, PUL, partial unilateral lentiginosis

A 13-year-old girl in good health presented with a cluster of pigmented lesions on the body that had been present since childhood. Upon examination, multiple brown, evenly pigmented macules were found to be present in the right scapular region, with sharp delineation at the midline (Fig 1); additionally, a few pigmented macules were found on the right buccal cheek (Fig 2) as well as right axillary freckling (Fig 3).

**Fig 1:**
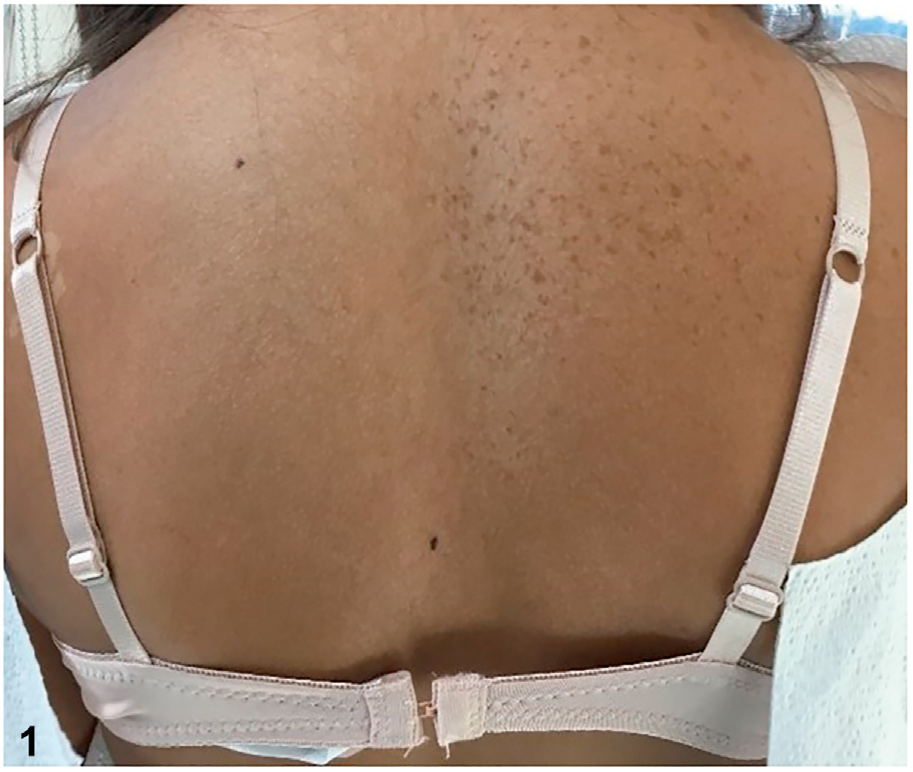

**Fig 2:**
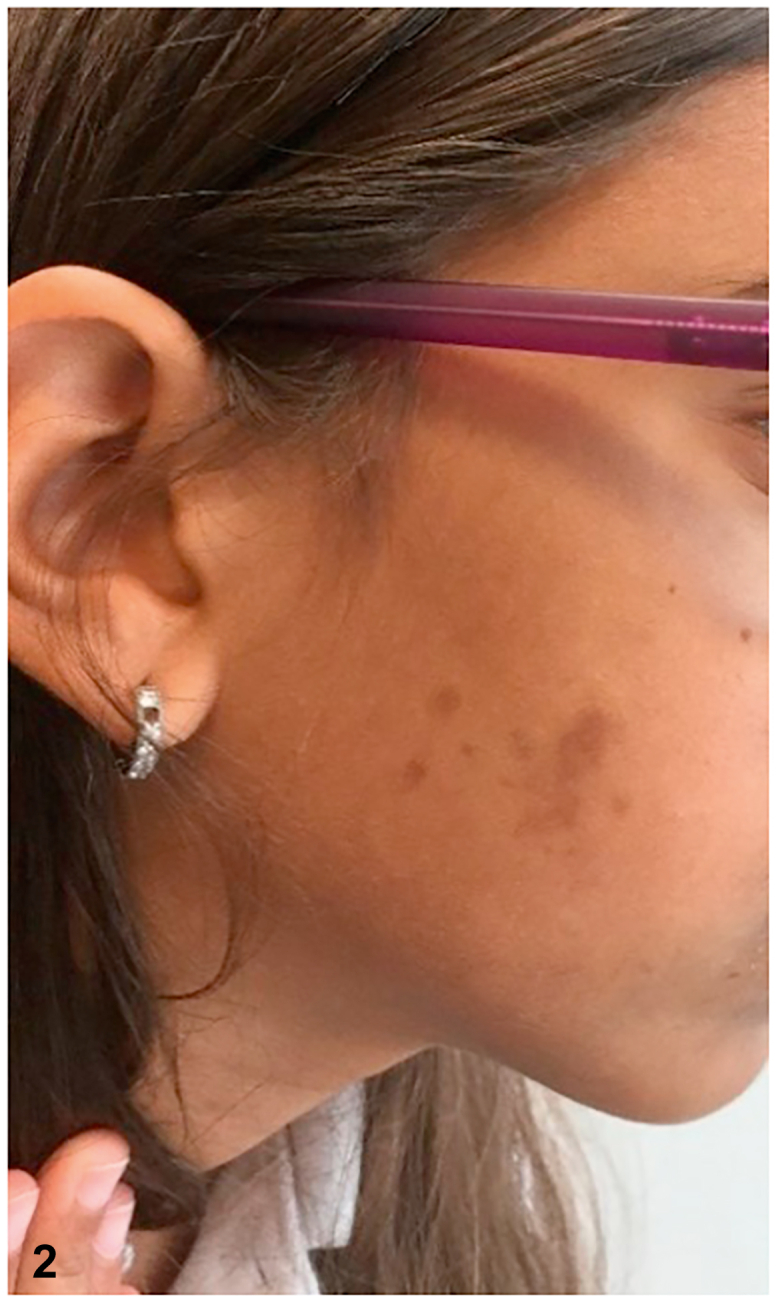

**Fig 3:**
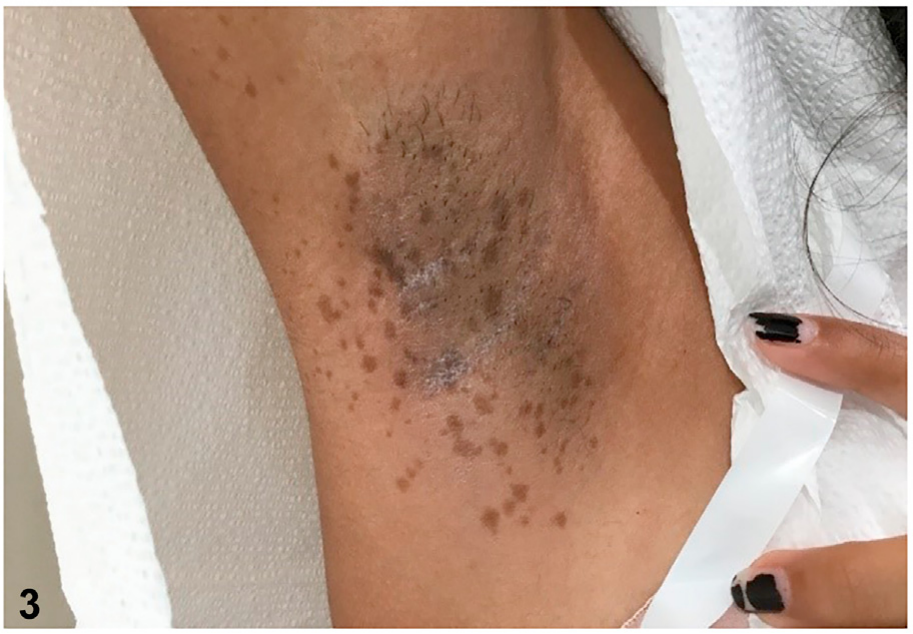

**Question 1: What is the likely diagnosis in this patient?**

A. Lentigo solaris
B. Partial unilateral lentiginosis (PUL)
C. Nevus of Ito
D. Speckled lentiginous nevus
E. Zosteriform lentiginous nevus

**Answers:**

A. Lentigo solaris – Incorrect. Lentigo solaris, also known as solar lentigo, is characterized by lentigines present on sun-exposed skin and is more commonly seen in older individuals. Histologically, there may be an increased number of melanocytes producing excess melanin, elongation of rete ridges, and solar elastosis within the dermis. This is in contrast to partial unilateral lentiginosis (PUL), which would look similar to lentigo simplex histologically; however, differences may arise in patient age, the clinical distribution of lesions, and histologic findings of solar elastosis.^1^
B. Partial unilateral lentiginosis (PUL) – Correct. PUL is a rare disorder of aberrant pigmentation characterized by multiple lentigines with a unilateral segmental pattern and may follow ≥1 dermatomal distributions. These lesions typically present at birth or during childhood, becoming darker and more widespread with age.^2^ The presence of lentigines in a distinctive distribution indicates the need for a thorough physical examination to rule out any associated syndromes, including but not limited to neurofibromatosis type 1 (NF1), LEOPARD (Lentigines, Electrocardiographic conduction defects, Ocular hypertelorism, Pulmonary stenosis, Abnormalities of the genitals, Retarded growth, Deafness) syndrome, and Carney complex.
C. Nevus of Ito – Incorrect. Nevus of Ito is a pigmented patch that is blue to gray-brown in color and is unilaterally located on the neck and shoulder. These nevi are typically present at birth and may mimic Mongolian spots.^1^
D. Speckled lentiginous nevus – Incorrect. Speckled lentiginous nevus, also known as nevus spilus, appears as small, dark macules superimposed on a lighter pigmented patch. PUL may commonly be misdiagnosed as nevus spilus; however, PUL appears as brown macules on normal skin, without an associated background of lighter pigmentation.^2^
E. Zosteriform lentiginous nevus – Incorrect. Zosteriform lentiginous nevus is from the family of nevus spilus; however, these lesions have more of a dermatomal arrangement.^3^

**Question 2: All of the following syndromes are strongly associated with multiple lentigines except which of the following?**

A. NF1
B. Sturge-Weber syndrome
C. LEOPARD syndrome
D. Carney complex
E. Peutz-Jeghers syndrome

**Answers:**

A. NF1 – Incorrect. NF1 is associated with axillary freckling. Many authors have suggested that PUL is a form of mosaic NF because of its association with axillary freckling, ipsilateral Lisch nodules, café-au-lait macules, and skeletal abnormalities.^2^ However, most patients do not demonstrate any other anomalies.
B. Sturge-Weber syndrome – Correct. Sturge-Weber syndrome is a vascular disorder that is not associated with lentigines. Although this syndrome may have ocular or central nervous system findings, its characteristic cutaneous finding is a unilateral vascular malformation known as a port-wine stain.^4^ This can present at birth and vary from pink to red blanching patches in a trigeminal nerve distribution.^4^
C. LEOPARD syndrome – Incorrect. LEOPARD syndrome, also known as multiple lentigines syndrome, describes the association of lentigines, electrocardiographic abnormalities, ocular hypertelorism, pulmonary stenosis, abnormalities of the genitalia, mental retardation, and sensorineural deafness.^5^
D. Carney complex – Incorrect. Carney complex, also known as nevi, atrial myxoma, myxoid neurofibroma, ephelides syndrome and lentigines, atrial myxoma, mucocutaneous myxoma, blue nevi syndrome, is characterized by the presence of mucocutaneous lesions, cardiac myxomas, and endocrine and nonendocrine tumors.^5^ Although its cutaneous findings have a predilection for eyelids and external ear canals, they may affect any part of the skin.^5^
E. Peutz-Jeghers syndrome – Incorrect. Peutz-Jeghers syndrome is an autosomal dominant syndrome with a hallmark finding of multiple, pigmented macules on the lips that present during childhood.^5^ These mucocutaneous findings may also be associated with gastrointestinal hamartomatous polyps and nongastrointestinal neoplasms such as endocrine tumors and thyroid nodules.^5^

**Question 3: What additional workup should be completed for this patient?**

A. Ophthalmologic examination
B. Infectious disease consultation
C. Autoimmune workup
D. Punch biopsy
E. Oncology consultation

**Answers:**

A. Ophthalmologic examination – Correct. PUL has been shown to be associated with a variety of ophthalmologic findings, including ipsilateral Lisch nodules, which are found in patients with NF1.^2^ In patients with PUL, a thorough physical examination is crucial, with focus on ophthalmologic and neurologic examinations to rule out any neurocutaneous syndromes.
B. Infectious disease consultation – Incorrect. There have been no reports of infection associated with PUL.
C. Autoimmune workup – Incorrect. There have been no reports of autoimmune disease associated with PUL.
D. Punch biopsy – Incorrect. A punch biopsy would be nondiagnostic of PUL because this may resemble lentigo simplex histologically.^1^
E. Oncology consultation – There have been no reports of malignant transformation of a lentiginous lesion in patients with PUL.

## Conflicts of interest

None disclosed.

## References

[1] Nieuweboer-Krobotova L. (2013). Hyperpigmentation: types, diagnostics, and targeted treatment options. J Eur Acad Dermatol Venereol.

[2] Kim H.T., Choi M.E., Na H. (2021). Partial unilateral lentiginosis: a clinicopathological analysis of 32 cases on the head and neck area in Korea. Int J Dermatol.

[3] Trattner A., Metzker A. (1993). Partial unilateral lentiginosis. J Am Acad Dermatol.

[4] Klar N., Cohen B., Lin D.D. (2016). Neurocutaneous syndromes. Handb Clin Neurol.

[5] Stratakis A. (2000). Genetics of Peutz-Jeghers syndrome, Carney complex and other familial lentiginoses. Horm Res.

